# Histological Analysis of SLC38A6 (SNAT6) Expression in Mouse Brain Shows Selective Expression in Excitatory Neurons with High Expression in the Synapses

**DOI:** 10.1371/journal.pone.0095438

**Published:** 2014-04-21

**Authors:** Sonchita Bagchi, Hajar Ali Baomar, Somar Al-Walai, Saifaddin Al-Sadi, Robert Fredriksson

**Affiliations:** Department of Neuroscience, Functional Pharmacology, Uppsala University, Uppsala, Sweden; University of South Florida, United States of America

## Abstract

SLC38A6 is one of the newly found members of the solute carrier 38 family consisting of total 11 members, of which only 6 have been characterized so far. Being the only glutamine transporter family expressed in the brain, this family of proteins are most probably involved in the regulation of the glutamate-glutamine cycle, responsible for preventing excitotoxicity. We used immunohistochemistry to show that SLC38A6 is primarily expressed in excitatory neurons and is not expressed in the astrocytes. Using proximity ligation assay, we have quantified the interactions of this SLC38 family protein with other proteins with known localization in the cells, showing that this transporter is expressed at the synapses. Moreover, this study has enabled us to come up with a model suggesting sub-cellular localization of SLC38A6 at the synaptic membrane of the excitatory neurons.

## Introduction

Among the membrane proteins that make up around 27% of all proteins in the human genome [1], the solute carriers are the second largest family with at least 395 members in human [2]. The solute carriers, also known as SLCs, are responsible for uptake and flow of several substances including amino acids, nucleotides, sugar, inorganic ions, and drugs across the cell membrane. It has been suggested that humans have almost 100 SLCs transporting amino acids [3]; 60% of which are confirmed to transport amino acids while the rest are phylogenetically most closely related to known amino acid transporters.

In mammals, SLCs are categorized into four phylogenetic clusters, namely α, β, γ and δ, based on phylogenetic analysis. The second largest cluster of amino acid transporters is the β group [3] that contains the SLC32 [4], SLC36 [5] and SLC38 families [6]. Most of the proteins of the β group are expressed in brain [7] and many of them appear to be located in the plasma membrane [8] and those that have been characterized regarding substrate specificity all transport for glutamine as well as a few other amino acids [9]–[12].

The SLC38 family consists of 11 members and the five that were first discovered, SLC38A1-SLC38A5, are relatively well-characterized [9], [13]–[18]. SLC38A1-7 proteins have been named sodium-coupled amino acid transporters (SNATs) [8], although SLC38A6 (SNAT6) is actually still an orphan transporter with unknown substrate profile. Furthermore, based on their substrate recognition profiles and biochemical properties, SLC38A1 [9], [13], SLC38A2 [14]–[16] and SLC38A4 [17] have been described as System A transporters, whereas, SLC38A3, SLC38A5 [10], [18] and most likely SLC38A7 [12] belong to the group of System N transporters. SLC38A6 [19] and the rest of the orphan members of this family have not been classified according to the N/A systems so far, as no substrate recognition profile has been reported for them yet.

As L-Glutamine appears to be a favored substrate for the SNAT family, this has led to the suggestion that these transporters are involved in the glutamine-glutamate cycle in the CNS [10], [18], [20], [21]. It is well established that SNAT3 is responsible for the uptake of glutamine into astrocytes [22] and it is generally believed that glutamine uptake into neurons is controlled by System A transporters expressed in the synapses of neurons [23]. It was suggested that SNAT1 and SNAT2 are responsible for this uptake, but an extensive electron microscopy study [23] has revealed that at most 1% of the SNAT1 and SNAT2 expression is found in the synaptic terminals.

Because the SLC38 transporters are the only known glutamine transporters, they are likely to be responsible for the entire glutamine metabolism of the brain [3]. One intriguing question that has arisen is what the functional differences between these large numbers of proteins with seemingly similar function are. One possibility is that there are differences regarding gene regulation, details in expression pattern or sub-cellular localization. Another possibility is that some of the SLC38 members function in dimers.

In this study, we report cellular localization of SLC38A6 transporter in the mouse brain. It was found to be expressed primarily in the glutamatergic neurons in brain. No expression of SLC38A6 has been found in astrocytes or glial cells. Based on protein-protein interaction analysis using proximity ligation assay (PLA), we propose a model to visualize the expression of SLC38A6 near the cellular membrane in relation to other proteins in its proximity.

## Experimental Procedures

### Ethical Statement

Animal care procedures for C57Bl6/J adult male mice were approved by the Uppsala ethical committee and followed the guidelines of European Communities Council Directive (86/609/EEC).

### Tissue Collection and Sectioning

All animal procedures were carried out in accordance with local regulations and followed the description in the approved ethical permit. Adult male C57Bl6/J mice (Taconic M&B, Denmark) were intra-peritoneally injected with pentobarbital (90 mg/kg IP; Apoteksbolaget, Sweden). The trans-cardial perfusion was performed through the left ventricle with phosphate-buffered saline (PBS) followed by 4% formaldehyde (HistoLab, Sweden). The brain was excised and stored in 4% formaldehyde overnight. The brain was then fixed in zinc-formalin (Richard-Allan Scientific) for 18–24 h at 40°C before dehydration and paraffin infusion (Tissue-Tek vacuum infiltration processor; Miles Scientific). The sections were cut (7 µm) using a Microm 355S STS cool cut microtome and attached on Superfrost Plus slides (Menzel-Gläser, Germany). Then each slide was dried overnight at 37°C and stored at 4°C until use.

### Fluorescent Immunohistochemistry on Paraffin Embedded Sections

Flourescent immunohistochemistry was performed according to the procedures described in [12], with some exceptions. Sections were incubated with the commercial polyclonal antibody rabbit-anti-SLC38A6 (Sigma-Aldrich HPA018508) together with one of the antibody markers (NeuN, GFAP, and PAG) diluted in supermix (Tris-buffered saline, 0.25% gelatin, 0.5% Triton X-100) overnight at 4°C (for antibody information see Table 1). After secondary antibody treatments (See Table 1 for concentrations) for 1 h and incubation with DAPI (Sigma-Aldrich, USA), the sections were mounted. Then sections were photographed using a Zeiss AxioPlan 2 fluorescence microscope, connected to an AxioCamHRm camera and the micrographs were finally analyzed with Carl Zeiss AxioVision version 4.8 software.

**Table 1:** Details of antibodies used for fluorescent immunohistochemistry and Proximity Ligation Assay.

**Primary antibodies**	**Species**	**Dilution**	**Company**
SLC38A6	Rabbit	1∶200	Sigma-Aldrich, USA
NeuN	Mouse	1∶400	Millipore, Sweden
Synaptophysin	Mouse	1∶250	BD Transduction lab, Sweden
GFAP	Chicken	1∶400	AbCam, United Kingdom
PAG	Mouse	1∶100	AbCam, United Kingdom
Synaptotagmin	Rabbit	1∶100	AbCam, United Kingdom
Synaptotagmin	Mouse	1∶100	Millipore, Sweden
Snap-25	Mouse	1∶500	Millipore, Sweden
Snap-25	Goat	1∶500	AbCam, United Kingdom
**Secondary antibodies**	**Species**	**Dilution**	**Company**
Anti-rabbit-488	Donkey	1∶400	Invitrogen, USA
Anti-mouse-594	Goat	1∶400	Invitrogen, USA
Anti-chicken-594	Goat	1∶400	Invitrogen, USA

### Cell Culture

The immortalized embryonic mouse hypothalamus cell line N25/2 (mHypoE-N25/2, CellutionsBiosystems Inc., Canada) was cultured in Dulbecco's Modified Eagle Medium (DMEM [+] 4.5 g/L D-Glucose, [+] L-Glutamine, [+] Pyruvate) from Gibco, Life technologies supplemented with 50 ml fetal bovine serum (FBS) (Gibco, Life technologies), 5 ml Penicillin-Streptomycin (Pen-Strep) (Gibco, Life technologies) and 5 ml amphotericin B (Gibco, Life technologies). All cells were incubated at 37°C with 5% CO_2_. The cells were seeded on glass slides (coated with 10 µg/ml poly-L-lycine) for 40 hours for prior to immuno-staining.

### Primary Cell Culture

C57Bl6/J female mice (Taconic M&B, Denmark) were mated with VIAAT- eGFP heterozygous males and at embryonic day 15–16, the females were sacrificed by severing the spinal cord. Embryos were removed from the uterus and kept in cold HBSS buffer (Gibco, Stockholm, Sweden) during separation from the yolk sac and placenta. Embryos were decapitated before cortex dissection was performed in 1x phosphate-buffered saline (PBS) with 10 mM glucose, under a Leica CLS 100 LED microscope. The cortices were chemically dissociated in 10 µg/ml DNase (Invitrogen, Stockholm, Sweden) and 0.5 mg/ml Papain (Sigma-Aldrich, Stockholm, Sweden), diluted in PBS with 10 mM glucose for 30 min at 37°C in presence of 5% CO_2_. Tissues were then rinsed in plating media DMEM-F12 (Gibco, Stockholm, Sweden) containing 10% FBS (Gibco, Stockholm, Sweden), 2 mM L-glutamine (Invitrogen, Stockholm, Sweden), 1 mM Na-Pyruvate (Invitrogen, Stockholm, Sweden) and 1% penicillin/streptomycin (Invitrogen, Stockholm, Sweden). Afterwards they were mechanically dissociated by pipetting up and down with a glass Pasteur pipette and filtered through a 70 µm nylon cell strainer (BD Stockholm, Sweden) to remove remaining cell clusters. Finally, the cells were plated at a density of 7.5*10^4^ cells on Poly-L-lysine (Sigma-Aldrich, Stockholm, Sweden) coated cover slips (12 mm, #1.5, Menzel-Gläser) and incubated for 2 h at 37°C in presence of 5% CO_2_. Plating media was then replaced with growth media Neurobasal-A (Gibco, Stockholm, Sweden) with 2 mM L-Glutamine, 1 mM Na-Pyruvate, 1% penicillin/streptomycin and 2% B27 (Invitrogen, Stockholm, Sweden). Two third of the growth media was changed every third day and on tenth day cells were rinsed with 37°C PBS with 10 mM glucose and fixated in 4% formaldehyde (Histolab, Sweden) for 10 min, followed by additional washes in PBS. Cells were kept in PBS until used.

### Fluorescent Immunohistochemistry on Cell-Line

In certain experiments, the plasma membrane of the cells were stained with 2 µg/ml fluorescently labeled Wheat Germ Agglutinin (WGA, Life technologies) prior to fixation. Cells were rinsed with PBS and fixed in 4% paraformaldehyde (Sigma-Aldrich, USA) for 15 min. Then the slides were pre-blocked with supermix (Tris-buffered saline, 0.25% gelatin, 0.5% Triton X-100) for 1 h at room temperature. Then the primary antibodies diluted in supermix were added to respective slides for overnight incubation at 4°C. After repeated washings with 1X PBS (Sodium perborate), they were incubated with secondary antibody (always 1∶400 dilutions in supermix when Alexa fluor is used, see Table 1 for details) for 1 h at room temperature. After several washing steps, DAPI (1∶2500 in PBS) was added for 10 min at room temperature. After washing, the slides were mounted in DTG media (with antifade (diazabicyclo (2.2.2) octane in 80% glycerol and 50 mM Tris pH 8.6) and photographed using a Zeiss AxioPlan 2 fluorescence microscope, connected to an AxioCamHRm camera. The micrographs were finally analyzed with Carl Zeiss AxioVision version 4.8 software.

### Proximity Ligation Assay (PLA)

The Duolink II fluorescence kit (orange detection reagents, Olink Biosciences, Sweden) was used to run *in situ* proximity ligation assay technology (PLA) on fixed cells and/or paraffin embedded sections (as described before) according to manufacturer's instructions [24]–[27]. The samples were blocked with blocking solution included in the kit for 30 minutes at 37°C in pre-heated humidity chamber. Then specific primary antibodies (see Table 1 for concentrations) diluted in antibody diluent included in the kit were added to the samples and incubated overnight at 4°C in humid chamber. After that PLA probes (PLUS and MINUS) were added for 1 hour at 37°C in pre-heated humidity chamber. In our experiments, protein interactions were detected with combinations of anti-rabbit PLUS and anti-mouse MINUS or anti-mouse PLUS and anti-goat MINUS PLA probes. Then the detection protocol including ligation and amplification was followed. The ligation step, using ligase (provided in the kit) was performed for 30 minutes at 37°C in pre-heated humidity chamber. The amplification step using polymerase (provided in the kit) was performed for 100 minutes at 37°C in pre-heated humidity chamber. Then the slides were washed according to the manufacturer's instructions with buffer provided in the kit, dried in the dark for 20 minutes and mounted using a minimal volume of Duolink *In Situ* Mounting Medium containing DAPI. The edges were sealed using transparent nail polish and were visualized using Zeiss Axioplan2 fluorescent microscope connected to AxioCamHRm camera. A negative control was included without primary antibodies. The images were further analyzed and quantified using Duolink ImageTool (Olink Biosciences, Sweden) software.

### Image Analysis

Z-stack images from different fields of the each slide were taken and PLA signals were counted with Duolink ImageTool (Olink Biosciences, Sweden) software. The average signal per cell was calculated and presented with 95% confident interval. The results were compiled and statistical analysis was done in GraphPad prism 5 software.

### 2D Gel Analysis

Total protein was extracted from cells with 80–100% confluency following the handbook 2-D electrophoresis; principles and methods by GE Healthcare. We used 2D fractionation kit (GE Healthcare) to lyse the cells and separate them into soluble and insoluble fractions. The cells were homogenized and all the steps were followed as directed by the manual with the kit. Further the samples were purified by 2D clean up kit (GE Healthcare) to get rid of lipids and nucleic acids from protein samples. Then the samples were resuspended in rehydration solution containing 8 M Urea, 4% Chaps, 20 mM DTT, 0,5% IPG Buffer (3–11), centrifuged for 5minutes at 12000 rpm and applied directly to Immobiline DryStrip gel (24 cm pH 3-11NL). The gels were then rehydrated for 12 hours (rehydration loading). The First-Dimension isoelectrical focusing step using EttanIGPhor II platform was as follows: 50 uA/strip, at 20°C. Voltage step and hold mode, 500 V 1 h; 1000 V 1 h; 8000 V to a total of 64 kVh. The Second-Dimension SDS-PAGE using Multiphor II Electrophoresis System included equilibration of IPG strips in 2% SDS, 50 mM Tris-HCL pH 8.8, 30% Glycerol, 0.002% BFB (15 min 10 ml+100 mg DTT and 15 min 10 ml+250 mg IAA). Each strip was placed on an ExcelGel XL 12–14% and the electrophoresis was performed at 15°C. Finally the gels were fixed in 10% MeOH and 7.5% Hac for 30 min.

### Western Blot

Western blot analysis was performed on Immobilon-P PVDF membrane (Millipore) from the 2D gel (described above) in order to detect SLC38A6 protein in cellular fractions. The proteins were pre-blocked for 1 h in blocking buffer (5% nonfat dry milk (Bio-Rad) diluted in 1.5 M NaCl, 0.1 M Tris, 0.05% Tween 20, pH 8.0). Then, the membrane was hybridized with the primary antibody against SLC38A6 (diluted 1∶200) overnight at 4°C. After washes in water, the membranes were incubated for 1 h with horseradish peroxidase-conjugated secondary antibody (diluted 1∶10000, goat anti-rabbit, Invitrogen) followed by detection with the enhanced chemi-luminescent (ECL) method. The membranes were incubated for 3 min in a 1∶1 mixture of luminol/enhancer and peroxidase buffer solutions (Immun-Star HRP, Bio-Rad) and developed on high performance chemi-luminescence film (GE Healthcare).

### Statistical Analysis

All statistical analysis and calculations have been done using software GraphPad Prism 5.

## Results

### Detection of SLC38A6 as a Membrane Protein

**Figure 1:**
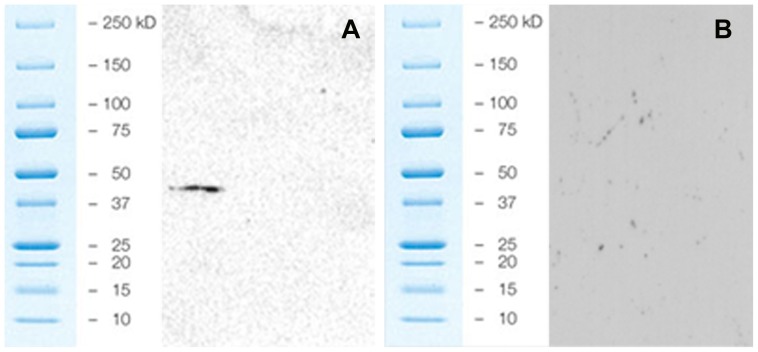
2D gel separation and blotting with SLC38A6 antibody of the total protein in mouse brain. A) Insoluble fraction of total protein indicating presence of SLC38A6 with the specific band between 37 and 50 KD. B) Soluble fraction of total protein indicating absence of SLC38A6 with no bands. No unspecific bands were detected in any of the fractions, validating the specificity of the antibody.

Total protein was extracted from whole brain of wild type mice and then separated into insoluble and soluble fractions. 2D gel analyses on these two fractions were performed separately. After blotting, both gels were hybridized with SLC38A6 antibody. As expected, we only found SLC38A6 protein in the insoluble fraction (Figure 1A) showing that SLC38A6 protein is indeed expressed on membranes. The size was between 37 and 50 kDa which agrees with the theoretical size of 50 kDa. As the antibody only gave one single band of the expected size in the membrane fraction and no SLC38A6 protein was detected in the soluble cytosolic fraction (Figure 1B), this strongly suggests that our SLC38A6 antibody is specific.

### Immunofluorescence on Paraffin-Embedded Brain Tissue Sections

**Figure 2:**
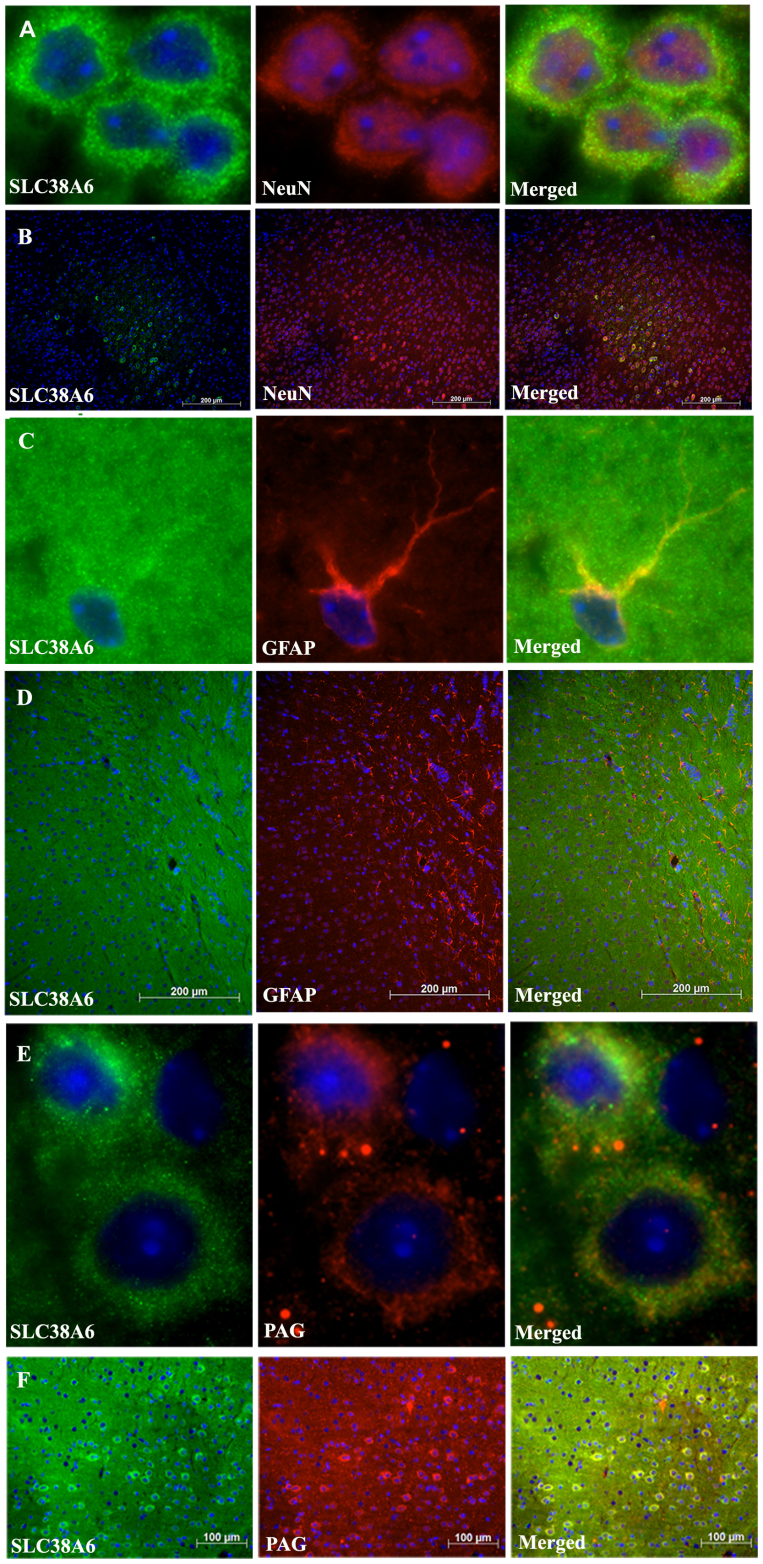
Fluorescence micrograph of immuno-labelling on paraffin-embedded mouse brain tissues (7 mm thickness) with 40X and 10X magnifications, respectively. In all panels, the nucleus is stained with DAPI in blue: A) Co-localization of SLC38A6 and NeuN is demonstrated with 40X magnification, where SLC38A6 is detected in green and the neural cells are stained in red. B) Co-localization between SLC38A6 and NeuN is further demonstrated with 10X magnification. C) No co-localization between SLC38A6 and glial cells/astrocytes is observed. SLC38A6 is detected at 40X magnification in green and glial cells are stained in red. D) No overlap between SLC38A6 and GFAP was recorded at 10X magnification. E) Expression of SLC38A6 in glutamatergic interneurons is illustrated at 40X magnification where SLC38A6 is detected in green and PAG is labeled in red. F) The overlap between SLC38A6 and PAG is demonstrated at 10X magnification.

We used immunohistochemistry with this SLC38A6 antibody and a number of antibody markers to identify the cell types expressing SLC38A6, shown in Figure 2. SLC38A6 co-localized partially with the neuronal marker NeuN [28] showing that SLC38A6 protein has higher expression in some neurons than others (Figure 2A with higher magnification and 2B with lower magnification). However, expression of SLC38A6 seems to be restricted to neurons as double immunohistochemical staining with SLC38A6 antibody and antibody against glial fibrillary acidic protein (GFAP), a marker for glial cells [29], showed no overlap (Figure 2C with higher magnification and 2D with lower magnification). To further specify the type of neurons with SLC38 expression, double-labeling was performed together with phosphate-activated glutaminase (PAG). PAG is an enzyme which generates glutamate and ammonia from glutamine and is a marker for glutamatergic neurons [30], [31]. Extensive overlap between SLC38A6 and PAG was observed showing that SLC38A6 is highly expressed in glutamatergic neurons (Figure 2E with higher magnification and 2F with lower magnification).

### High Resolution Confocal Micrograph and Proximity Ligation Assay Confirms No Expression of SLC38A6 in Glial Cells

**Figure 3:**
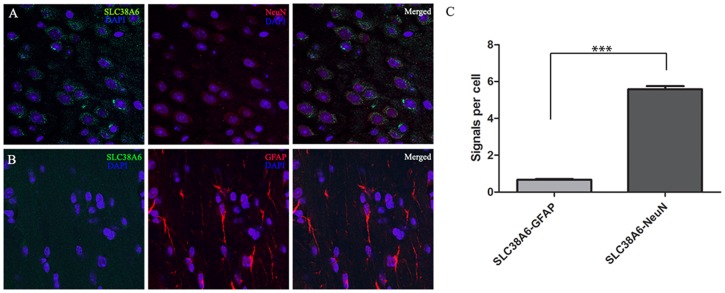
Confocal micrograph (63 times magnification) and PLA data confirming significantly higher expression of SLC38A6 in the neurons compared to the glial cells on paraffin-embedded tissue sections of whole mouse brain. A) Three panels illustrate SLC38A6 staining in green (DAPI in blue), NeuN in red (DAPI in blue) and three color overlay (merged), respectively. B) Three panels here represent SLC38A6 staining in green (DAPI in blue), GFAP in red (DAPI in blue) and three color overlay (merged), respectively. C) Quantification of PLA signals per cell exhibits higher interaction between SLC38A6 and NeuN (neuronal marker) than that between the transporter and GFAP (glial cell marker), confirming no or minor expression of SLC38A6 in the glial cells compared in the neurons. Statistical analysis (t test) shows that interaction between SLC38A6 and NeuN is significantly higher than that between SLC38A6 and GFAP.

Our confocal micrograph at 63 times magnification on paraffin-embedded tissue sections revealed overlap between SLC38A6 and neural marker NeuN [28], whereas there was no obvious co-localization observed between the former and glial cell marker GFAP [29] (Figure 3A and 3B, respectively). To confirm further that the transporter is expressed exclusively in the neurons, we used Proximity Ligation Assay (PLA). PLA specifically detects proximity between two proteins with maximum spatial distance of 40 nm. We have performed PLA on paraffin-embedded tissue sections between SLC38A6 and NeuN as well as SLC38A6 and GFAP. After quantification of PLA signals per cell, we concluded from the histograms (Figure 3C) that the transporter interacts approximately 10 times more with the neural marker compared to that of the glial cell marker. Statistical analysis (t test) on the data set showed significant (P<0.0001) difference between positive signals recorded by PLA between SLC38A6-GFAP and SLC38A6-NeuN, confirming significantly higher expression of SLC38A6 in neurons compared to that in glial cells.

### Immunofluorescence on Primary Cell Culture

**Figure 4:**
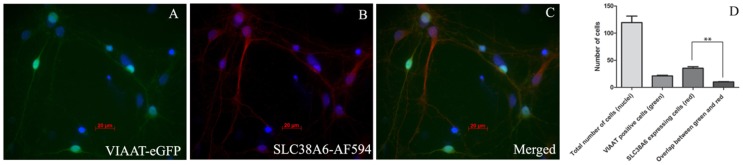
Fluorescence micrograph of immunohistochemistry executed on primary cell culture of mouse embryo cortex at 20X magnification. A) VIAAT or VGAT protein tagged with eGFP is viewed in green and the nucleus is stained with DAPI in blue. B) SLC38A6 protein is visualized with Alexa-flour 594 (AF594) secondary antibody in 1∶400 dilution in red and the nucleus is stained with DAPI in blue. C) Merged image of VIAAT/VGAT-eGFP in green and SLC38A6-AF594 in red together with the nucleus, stained with DAPI in blue. D) Quantification of VIAAT/VGAT positive cells and SLC38A6-expressing cells and their overlap in comparison with total number of cells in each tissue section. The first column exhibits total number of cells, whereas the second and third column represents VIAAT/VGAT positive (green signals) and SLC38A6-expressing (red signals) cells, respectively. The last column shows overlap between green and red signals, representing the cells expressing both VIAAT/VGAT and SLC38A6 proteins. The number of cells counted was 1193 (in total). Statistical analysis (1way ANOVA) exhibits significantly higher number of cells expressing SLC38A6 compared to cells with overlapping red and green signals.

We used cells from primary cell culture where the inhibitory neurons are marked with eGFP (Figure 4A) and stained them for SLC38A6 by Immuno-histo chemistry (Figure 4B) with Alexafluor 594. Interestingly, the transporter was found to be much less expressed in the inhibitory neurons as there was not much overlap (Figure 4C) between green fluorescence from eGFP fused VGAT or VIAAT protein [8] and the red fluorescence from secondary antibody (Alexafluor 594) used to bind to specific primary antibody SLC38A6. We have analysed the micrographs by counting number of cells using Carl Zeiss AxioVision version 4.8 software. We have separately counted the cells exhibiting green and red flurescence as well as overlapping each other. We have also shown the total number of cells (nuclei) and have plotted all combinations (Figure 4D). This data shows overlap between green and red signals to some extent, but the eGFP positive cells with strong green signal often had quite weak red signal whereas cells with stronger red signal often were not exhibiting green signal at all. We have further performed 1way ANOVA on the data set and found significant difference between cells expressing SLC38A6 and cells having both green and red signals. This illustrates that the number of cells expressing SLC38A6 is significantly higher than the cells expressing both SLC38A6 and eGFP fused VGAT or VIAAT protein.

### Protein-Protein Interaction between SLC38A6 and Vesicular and Membrane Markers

**Figure 5:**
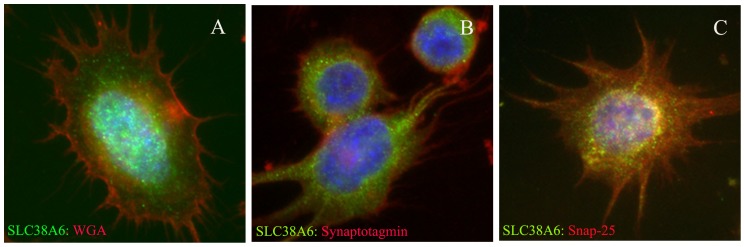
Fluorescence micrograph of N25/2 hypothalamic cell line with 40X magnification. Nucleus is stained blue with DAPI in all panels. A) Simultaneous localization of SLC38A6 (in green) and WGA (in red) is visualized. B) Localization of SLC38A6 (in green) in context with synaptotagmin (in red) is demonstrated here. C) Localization of SLC38A6 (in green) in relation with Snap25 (in red) is illustrated.

**Figure 6:**
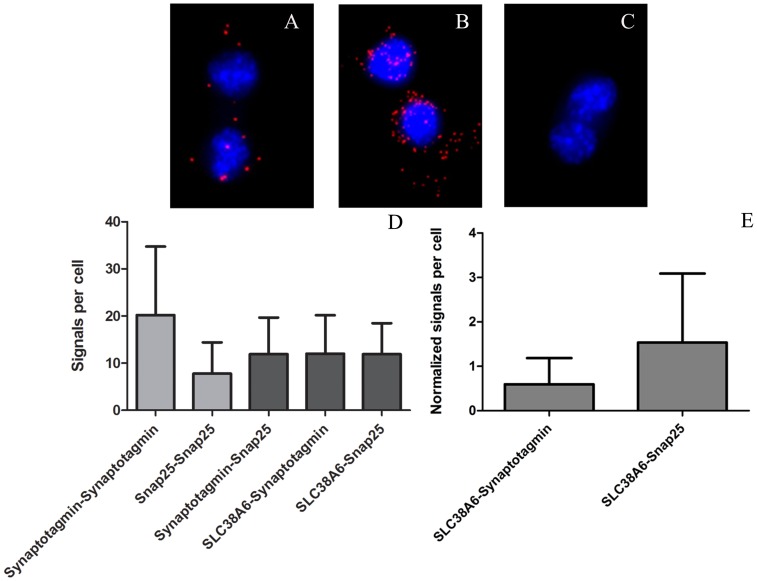
Proximity ligation assay (PLA) on N25/2 hypothalamic cell line to demonstrate protein-protein interaction pattern between SLC38A6 and neuronal markers. The nuclei of the cells are stained in blue with DAPI. The PLA signals are detected with red filter. A) SLC38A6 interacts with Snap25 exhibiting proximity to the membrane. B) SLC38A6 interacts with Synaptotagmin, illustrating substantial proximity to the vesicular proteins. C) PLA control with no primary antibody incubation and hence no signal of interaction between proteins demonstrating successful PLA in the treated cells. D) Signals per cell were counted and represented here as histograms. Columns 1, 2, 3, 4 and 5 reveal interactions between Synaptotagmin-Synaptotagmin, Snap25-Snap25, Synaptotagmin-Snap25, SLC38A6-Synaptotagmin and SLC38A6-Snap25, respectively. Columns 1 and 2 exhibit quantification of PLA signals obtained by using different antibodies on the same proteins, Synaptotagmin and Snap25 respectively. Column 3 depicts interaction between synaptotagmin and Snap25 to illustrate their proximity in the cells. Columns 4 and 5 show interactions between SLC38A6 and synaptotagmin and Snap25, respectively. E) Normalized signals per cell are shown here to compare interactions of SLC38A6 with Synaptotagmin and Snap25, respectively. Column 1 and column 2 demonstrate normalized interactions between SLC38A6 and synaptotagmin and SLC38A6 and Snap25, respectively.

Our results from double labelling indicated simultaneous expression of SLC38A6 and vesicular protein synaptotagmin as well as SLC38A6 and plasma membrane protein Snap25 (Figure 5B and 5C, respectively). Therefore, we further investigated the relationships between SLC38A6, Snap25 and synaptotagmin by Proximity Ligation Assay (PLA). PLA analysis revealed that SLC38A6 is in close proximity with the neural markers Snap25 and synaptotagmin (Figure 6A and 6B, respectively). Figure 6C is the negative control with no signal in absence of primary antibody. Statistics on our PLA data showed that the numbers of proximity signals are almost same between SLC38A6 and Synaptotagmin and that of SLC38A6 and Snap25 (Figure 6D, columns 4 and 5, respectively). We further studied proximity between Snap25 and Synaptotagmin and as expected they appear to be located within 40 nm range to each other (Figure 6D, column 3). Furthermore, we performed PLA between synaptotagmin proteins and Snap25 proteins with antibodies raised against the same proteins in different species (Figure 6D, columns 1 and 2). Biologically, this data account for the measure of expression of synaptotagmin and Snap25, respectively, demonstrating that there is more detectable synaptotagmin (Figure 6D, column 1) compared to Snap25 (Figure 6D, column 2) in this cell line. We normalized the proximity data of SLC38A6 and synaptotagmin as well as the proximity data of SLC38A6 and Snap25 with the numbers calculated from synaptotagmin-synaptotagmin and Snap25-Snap25 assay. We divided the number of signals between SLC38A6 and synaptotagmin with that between synaptotagmin (raised in rabbit) and synaptotagmin (raised in mouse). Similarly, number of PLA signals between SLC38A6 and Snap25 was divided by that between Snap25 (raised in mouse) and Snap25 (raised in goat). Interestingly, the normalized data exhibited higher interaction of SLC38A6 with Snap25 compared to that of synaptotagmin (Figure 6E), suggesting higher proximity as well as probable interactions between the former pair compared to the latter.

## Discussion

It has previously been shown by *in situ* hybridization and quantitative realtime PCR (qRT-PCR) that SLC38A6 mRNA is widely expressed in mouse brain [7]. In this study, we used immunohistochemistry and proximity ligation assays to explore which specific cells in the mouse brain have SLC38A6 protein expression as well its precise subcellular localization.

Previously characterized SLC38 proteins have been found to localize either in the neurons or in astrocytes [10]–[12] or in both type of cells [9], [32], [33]. Our co-localization studies using immunohistological double labeling exhibited expression of SLC38A6 exclusively in neurons and not in astrocytes and glia cells (Figure 2A-D and Figure 3A and 3B). This specific expression pattern has been further confirmed by performing PLA between SLC38A6 and NeuN as well as SLC38A6 and GFAP (Figure 3C) and statistically (t test) showing significant difference between the interactions. We have also shown that SLC38A6 is co-localized with the marker for phosphate-activated glutaminase (PAG), an enzyme which generates glutamate and ammonia from glutamine in glutamatergic neurons (Figure 2E and 2F). We have failed to use GABAergic neural marker (e.g. the glutamic acid decarboxylase 67 protein marker) successfully in our experiment on paraffin-embedded tissue sections. However, we have effectively used primary cells, where VIAAT or VGAT protein is tagged to eGFP, and illustrated that SLC38A6 is less prominently expressed in the GABAergic inhibitory neurons compared to the other neuronal cells. This conclusion is strengthened by the observation that many of the overlaps we recorded had strong green signals but quite weak red signals, whereas the cells with strong red signals often had no green signal (Figure 4A–D). Statistical analysis (1way ANOVA) illustrates that the number of cells expressing SLC38A6 is significantly higher than the number of cells expressing both SLC38A6 and VIAAT or VGAT protein. We thus conclude that SLC38A6 is primarily expressed in excitatory neurons with some minor expression in the inhibitory neurons.

We have used a commercially available (Sigma-Aldrich) antibody raised against SLC38A6 for this study. Interestingly, it was not possible to confirm specificity of this antibody directly by western blot. We could only detect this protein when we separated the total protein by 2D gel procedure (separation between pH 3 to 11) and then blotted with SLC38A6 antibody. This might indicate masking of SLC38A6 by some complex formation, or this could also be due to presence of numerous proteins between 50 to 75 KD molecular weight in the total protein pool of wild type mouse brain. Nevertheless, this validates the specificity of the antibody. SLC38A6 is most likely a membrane protein, based on primary sequence data and phylogenetic analysis [3] and our 2D gel analysis with antibody blotting confirms presence of SLC38A6 in the insoluble fraction whereas no signal was detected in the soluble cytosolic fraction.

**Figure 7:**
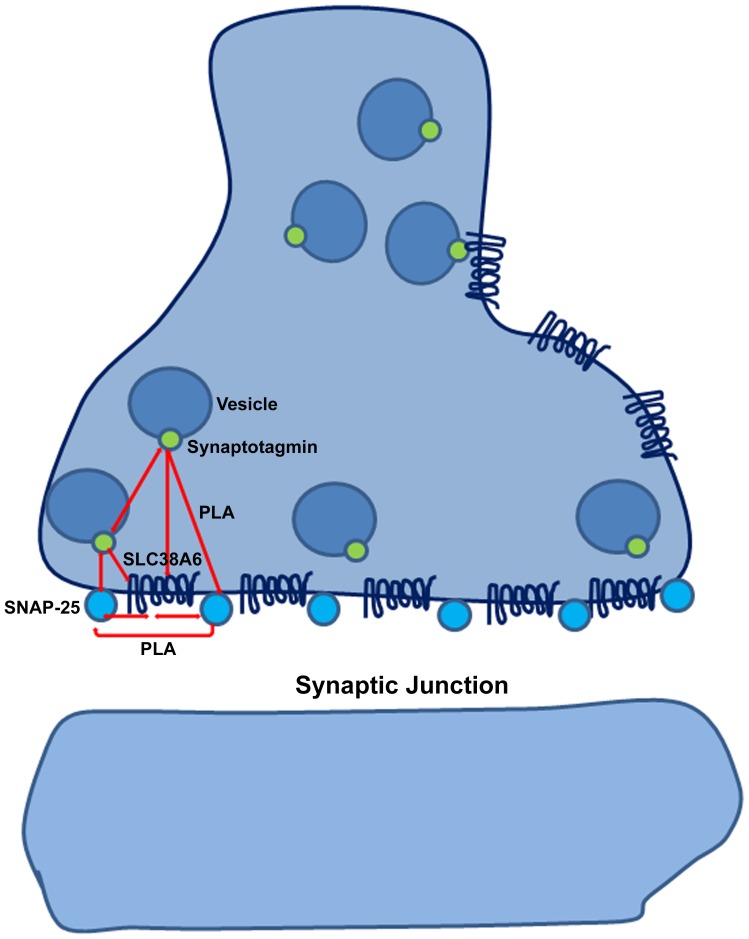
Schematic diagram of relative location of SLC38A6 in the cell. According to our proposed model, SLC38A6 is located at the membrane of synapse and is shown as a trans-membrane protein in dark blue. It interacts with the well-known membrane protein Snap25 present at the synaptic junction/cleft and is represented here as light blue circles. The vesicular protein Synaptotagmin is shown in green and is interacting with SLC38A6 when the vesicles are in close proximity to the membrane, prior to the release of neurotransmitters. Some of the relevant interactions, as recorded by PLA signals, are shown by red double arrows.

As SLC38A6 co-localized with Synaptotagmin and Snap25 in Immuno-histo chemistry experiment on N25/2 cells, Proximity Ligation Assay was performed to explore further the relationships between these three proteins by quantifying cellular proximity between them. We propose a model (Fig 7) here that can explain the interaction patterns of SLC38A6, Synaptotagmin and Snap-25, taking into account their relative localizations in the cell. Primarily, it is possible that even though SLC38A6 is present on the membrane, they are in close proximity with both membrane proteins and vesicular proteins in the cell membrane at a pre-synaptic cleft (Figure 7). The expression levels of different proteins are of immense importance here, as protein molecules, expressed in higher numbers would provide more interaction signals compared to that of less expressed ones. We therefore performed PLA with two antibodies raised in different species against the same protein to estimate the number of signals that can be detected with PLA for this specific protein. Our normalized data shows that the number of PLA signals per cell is much higher between SLC38A6 and Snap25 compared to that of SLC38A6 and Synaptotagmin. In column 3 in Figure 5, PLA between Synaptotagmin and Snap25 shows countable proximity between membrane proteins and vesicular proteins, demonstrating that there are certain vesicular proteins present in proximity of membrane proteins. PLA between SLC38A6 and synaptophysin, a ubiquitously expressed protein in the brain showed limited interaction signals per cell (Data not shown). This can be considered a positive control for our PLA experiment and exhibits the fact that SLC38A6 is not ubiquitously expressed in the brain, but rather has a specific location. This is also strengthened by the fact that SLC38A6 is found primarily in excitatory neurons, by using immunohistochemistry on primary culture of the mouse brain. As the glutamate/glutamine cycle shunts amino acids from extracellular space to astrocytes and then back into the excitatory neurons, our model suggests that SLC38A6 (among others) can be involved in transport of certain amino acids at the junctions during this advanced mechanism. SLC38A6 is not yet formally shown to be a glutamine transporter; however it is highly likely that it is a glutamine transporter based on the fact that it is most similar to SLC38A1-A5 [8]. SLC38A7 is also a glutamine transporter [12], but is very different in primary sequence from SLC38A1-A5 [8] and is still similar in substrate profile to SLC38A1-A5 [12].

It was originally suggested that SNAT1 and SNAT2 could play this role, although it was later shown that these have very limited expression on neuronal synapses in the brain [23]. SNAT1 and SNAT2 are the well characterized system A transporters and a hall mark for system A. One early study [34] also showed that blocking of System A transporters with MeAIB did not prevent glutamine uptake in the glutamate/glutamine cycle, showing that the neuronal component of this cycle must be accounted for by MeAIB insensitive transporters. The synaptic localization as well as the selective expression in excitatory neurons suggests that SLC38A6 can be functionally relevant as a neuronal transporter for the glutamate/glutamine cycle. However it remains to be shown what the substrate profile of SLC38A6 is regarding transport capacity for glutamine. Also it remains to be seen if this transporter is indeed MeAIB insensitive.

As observed in figure 4, our immune-histo chemistry data on primary cell culture shows presence of SLC38A6 along the dendrites (Figure 4A). This can be explained by assuming that SLC38A6 is expressed at the dendritic extensions as a backup. This could not be further explored in this article due to lack of consistent dendritic markers and availability of primary cells in our facility.

In conclusion, we have shown that SLC38A6 is primarily expressed in excitatory neurons and we also show that SLC38A6 is expressed in the synapse. The substrate profile of this transporter is yet to be determined, but its localization indicates significant possibility of its involvement in glutamate/glutamine cycle.
